# Modulation of brain activity and tremor severity by NeuroEpo in Parkinson’s patients with varying tremor amplitudes

**DOI:** 10.3389/fneur.2025.1637184

**Published:** 2025-10-01

**Authors:** Ahmad A. Jiman, Eyad Talal Attar

**Affiliations:** ^1^Department of Electrical and Computer Engineering, Faculty of Engineering, King Abdulaziz University, Jeddah, Saudi Arabia; ^2^Center of Excellence in Intelligent Engineering Systems (CEIES), King Abdulaziz University, Jeddah, Saudi Arabia; ^3^Center of Research Excellence in Renewable Energy and Power Systems, King Abdulaziz University, Jeddah, Saudi Arabia

**Keywords:** Parkinson’s disease, NeuroEPO, tremor characteristics, motor function, cognitive function, deep brain stimulation (DBS), neuronal activity, high amplitude tremor (HAT)

## Abstract

**Background:**

Tremors, particularly those related to Parkinson’s disease (PD), significantly affect quality of life. In this study, we inspected the effects of NeuroEpo versus placebo on brain function and tremor inhibition in patients with high-amplitude tremors (HAT) and low-amplitude tremors (LAT).

**Objectives:**

To assess the efficacy of NeuroEpo in enhancing coherence and power spectral density (PSD) relative to tremor severity, and to explore differences in stimulation parameters and medication requirements between patients with HAT and LAT.

**Methods:**

A double-blind randomized controlled trial was conducted on 16 participants diagnosed with PD. Participants were stratified into the HAT (*n* = 8) and LAT (*n* = 8) groups. Deep brain stimulation (DBS) parameters were individualized, and participants received either NeuroEpo or a placebo. The study was approved by the IRP on October 2, 2001, and all research participants gave informed consent. This research was conducted at the University of Quebec at Montreal, McGill University in Montreal, Quebec, Canada, and CHU de Bordeaux at Hôpital du Haut-Lévêque, Pessac, France. Data were obtained from authentic online platforms using DOI-referenced sources. Outcome measures included tremor severity, brain activity via EEG, and medication dosage.

**Results:**

NeuroEpo treatment produced a significant increase in coherence and PSD, particularly in the 20–21 Hz frequency band, compared with placebo. Patients with LAT required higher stimulation intensities and medication doses than those with HAT, suggesting a more complex disease profile. The combination of NeuroEpo and DBS resulted in improved both motor and cognitive functions. A two-way ANOVA indicated a significant main effect of treatment (*p* < 0.05), with the NeuroEpo group exhibiting a significantly greater PSD than the placebo group.

**Conclusion:**

NeuroEpo may provide an adjuvant therapeutic aid for the control of tremor and improvement in brain function in PD patients. Customized treatment approaches incorporating tremor amplitude may improve therapeutic benefit and patient well-being. Future research should follow up on the long-term psychosocial effects of combined interventions.

## 1 Introduction

This study addresses a critical knowledge gap regarding the potential contribution of NeuroEpo to enhanced tremor control and cognition, particularly in Parkinson’s disease (PD) and other neurodegenerative disorders. Tremors, one of the most common movement disorders worldwide, are characterized by involuntary rhythmic movements that impair daily functioning and quality of life. While current therapies can be operative for some patients, they often fail to offer comprehensive symptom relief, especially for the cognitive and affective aspects of PD-related tremors. Accordingly, this research discovers NeuroEpo as an adjunct treatment to address such gaps and to complement traditional interventions such as deep brain stimulation (DBS) and pharmacotherapy (1).

PD and essential tremor—two prevalent neurodegenerative conditions—are both related to an age-related increase in tremor frequency (1). Tremors are classically classified as high-amplitude tremors (HAT) or low-amplitude tremors (LAT), which differ in clinical presentation and therapeutic responsiveness (2). HAT involves pronounced oscillatory movements that severely affect daily life, while LAT is less overt but still significantly reduces quality of life. Both types occur from dysfunction in the basal ganglia–thalamocortical circuitry, which regulates motor control (3). Imbalances in excitatory and inhibitory neurotransmission in these regions can lead to pathological neural oscillations. Increased activity in the subthalamic nucleus (STN) and globus pallidus interna (GPi) is involved in tremor generation, influencing treatment targets such as DBS (4). DBS, which distributes targeted electrical stimulation to the STN or GPi, has transformed tremor treatment, particularly in drug-resistant cases (5, 6).

The success of DBS varies between tremor type, disease duration, overall health, and the use of DBS also depends on patient groups. However, the benefit is only derived if optimal parameters of stimulation frequency (130–185 Hz), amplitude, and duration of pulses (~90 μs) are used. More tremor suppression is generally achieved at higher frequencies (7), and more effective symptomatic amelioration might be obtained from the intensification of the intensity (8). Despite its effectiveness, DBS responses vary extensively, creating a requirement for complementary treatments. Pharmacologic choices—including beta-blockers, anticholinergics, and dopaminergic agents—primarily offer symptomatic release.

LAT patients often need higher medication doses than HAT patients, suggesting distinct pathophysiological mechanisms and differences in responsiveness to both pharmacotherapy and DBS (2).

Neurophysiological studies specify that DBS not only reduces tremor severity but also enhances cortical–subcortical connectivity, improving both motor and cognitive function (9). This broader influence highlights the potential for NeuroEpo to augment DBS effects by further enhancing cognitive outcomes and addressing psychosocial aspects often overlooked in standard treatments (10). However, its use must be enhanced with individualized treatment parameters.

PD is a chronic, progressive neurodegenerative disorder noticeable by tremor, rigidity, and bradykinesia—symptoms that greatly diminish quality of life. DBS is well-established for PD patients with tremor who respond poorly to medication (11). Multiple studies have confirmed significant reductions in tremor severity and improvements in motor function with DBS (12, 13). Nonetheless, outcome variability and the potential essential for supplementary treatments, such as NeuroEpo, have prompted further investigation into combined therapeutic approaches. Evidence recommends that NeuroEpo can enhance brain activity modulation in PD, leading to improved clinical outcomes (14, 15). Table 1 summarizes key findings from previous DBS studies on tremor management in Parkinson’s disease.

**Table 1:** Comparison of the present study on NeuroEpo with previous research.

Study	Objective	Methodology	Findings	Conclusion
Katzenschlager et al. (2005) (11)	To evaluate the effectiveness of DBS in managing tremor symptoms in PD.	A randomized controlled trial involving 50 participants with Parkinson’s disease with random assignment to DBS or sham treatment	Significant reduction in tremor severity in the DBS group compared to control at 6-and 12-months post-treatment.	DBS is effective at managing tremors in Parkinson’s disease, leading to improved motor function and quality of life.
Dilek et al. (2018) (12)	To analyze the effects of DBS on tremor severity through a meta-analysis.	Meta-analysis of 10 randomized controlled trials assessing different DBS techniques and tremor assessments.	Significant reduction in tremor severity across studies, with varying outcomes based on stimulation targets and parameters.	DBS significantly alleviates tremor symptoms; however, optimal parameters may vary among individuals.
Kumar et al. (2010) (13)	To investigate the efficacy of DBS for tremor in Parkinson’s disease.	A prospective study involving 60 patients underwent DBS for tremor, assessing outcomes over 12 months.	Patients showed significant improvement in tremor scores and quality of life measures post-DBS.	DBS is a reliable intervention for the management of Parkinson’s disease-associated tremors, improving both symptoms and quality of life.
Follett et al. (2010) (14)	To compare the effectiveness of two DBS targets (GPi vs. STN) in PD.	Randomized trial of 298 participants assigned to receive either GPi or STN stimulation, with follow-up assessments.	No significant difference in overall tremor reduction, but differences in side effects were noted between targets.	Both the GPi and STN are effective targets for tremor management; the choice of target may depend on patient-specific factors.
Zhang et al. (2016) (15)	To investigate the neurophysiological changes associated with DBS.	EEG recordings and clinical assessments were performed on 30 patients both before and after DBS surgery.	Changes in brain oscillations correlated with tremor improvement post-DBS; specific frequency bands were notably affected.	Understanding neurophysiological changes post-DBS can help to optimize treatment strategies for tremor disorders.

Despite these improvements, substantial gaps remain—particularly regarding the long-term efficacy of DBS and pharmacotherapy, as well as the broader psychosocial impact of tremor disorders. The present study was designed to compare the differential effects of DBS in HAT and LAT patients by examining stimulation parameters, medication requirements, and neurophysiological outcomes. In doing so, it sought to determine whether NeuroEpo, as an adjunct to DBS, offers distinct benefits based on tremor amplitude. Given the variability in DBS outcomes and the limited research on combined neuroprotective and neuromodulatory strategies, the study investigates whether NeuroEpo differentially enhances neural connectivity and tremor control in HAT versus LAT groups (16).

## 2 Methods

### 2.1 Study design

This study was a randomized, double-blind, placebo-controlled trial that tested the effects of NeuroEpo compared to placebo on brain function and tremor in patients with high-amplitude tremors (HAT) and low-amplitude tremors (LAT). This study recruited participants from a clinical population with Parkinson’s disease (PD), grouping them by tremor amplitude: HAT and LAT. The data are from online sources (17).

Six-week follow-up from baseline and primary outcome measures at baseline, week 2, week 4 (end of treatment), and week 6 (2 weeks post-treatment). The IRP granted ethical approval for the protocol on 2 October 2001, and informed consent procedures were followed in all research activities. The study was conducted over three centers: the University of Quebec at Montreal, McGill University (Montreal, Quebec) and Hôpital Haut Leveque-Hopital Xavier Arnozan CHU de Bordeaux (Pessac, France).

### 2.2 Participants

A total of 16 participants joined—8 in the HAT group and 8 in the LAT group. Tremor classification was based on peak-to-peak rest tremor amplitude recorded from the index finger: > 4 mm was categorized as HAT, and ≤4 mm as LAT (18). Recordings were taken under conditions with and without DBS and medication.

Stimulation targeted one of three brain regions: the ventral intermediate nucleus of the thalamus (Vim), the internal globus pallidus (GPi), or the subthalamic nucleus (STN). Tremor velocity was measured using a low-intensity velocity-transducing laser. Each subject was tested under 55 recording conditions, including DBS on/off and medication on/off, with additional assessments at 15, 30, 45, and 60 min after stimulator arrest (18).

Inclusion criteria:

- Confirmed PD diagnosis
- Classification of tremor amplitude (HAT or LAT) by clinical assessment
- Stable medication regimen for at least one month before the study

Exclusion criteria:

- Other neurological disorders
- Participation in other clinical trials
- Contraindications to DBS or EEG monitoring

### 2.3 Demographic and clinical data

Collected data included age, sex, and disease duration (years since diagnosis). Clinical variables included stimulation target (GPi or Vim), stimulation parameters (effective and ineffective frequency, intensity, and pulse width), and medication dosage.

### 2.4 Stimulation protocol

Deep Brain Stimulation (DBS) parameters were individualized:

- Frequency: 130–185 Hz (effective)
- Ineffective frequency: 60–90 Hz (preconditioned)
- Intensity: 1.3–5.3 V
- Pulse width: 90 μs for optimal effect
- Configuration: Predominantly monopolar unless bipolar yielded superior clinical response; double monopolar settings were not used.

### 2.5 Randomization and blinding

Participants were randomly assigned to NeuroEpo or placebo using a computer-generated block randomization (block size = 4) performed by an independent party. Allocations were sealed in sequentially numbered opaque envelopes. Both participants and investigators remained blinded, and blinding integrity was verified at study completion—accuracy was at chance level.

### 2.6 Intervention

NeuroEpo was administered intranasally at a dose of 1.2 mg (≈3,000 IU) per session, divided into two daily doses over 4 weeks. Placebo consisted of isotonic saline with identical appearance, viscosity, and delivery method. Dose adjustments were permitted for tolerability. This regimen was based on prior PD trials (7, 19). Minor side effects reported in previous studies include transient nasal irritation and mild headache; no serious adverse events were anticipated.

### 2.7 Outcome measures

- Tremor Severity: UPDRS Part III (Motor Examination) items 20 (rest tremor amplitude) and 21 (action/postural tremor of hands).
- Brain Activity: EEG coherence and power spectral density (PSD) in the 9–12 Hz and 20–21 Hz frequency bands. EEG was performed at baseline and 60 min post-dose during weekly visits.
- Medication Dosage: Recorded to evaluate pharmacological management across groups.
- Quality of Life: Parkinson’s Disease Questionnaire (PDQ-39) and UPDRS-ADL subscale.

### 2.8 EEG recording and analysis

EEG was recorded using a 32-channel cap (sampling rate: 256 Hz).

1. Preprocessing: 0.5–50 Hz bandpass filter; artifact removal via Infomax ICA; re-referencing to the average of all electrodes.
2. Topographic Mapping: Visualized coherence and intensity across cortical regions.
3. Frequency Analysis: PSD computed for the 9–12 Hz and 20–21 Hz bands.

### 2.9 Statistical analysis

Power calculations indicated that 16 participants would provide 80% power to detect a moderate effect size (Cohen’s *d* = 0.5) at *α* = 0.05 (two-tailed). Analyses included:

- Descriptive statistics for demographics and baseline characteristics.
- Two-way ANOVA to compare treatment effects (NeuroEpo vs. placebo) on PSD across frequency bands.

The significance level (*α*) for confirmatory testing of the pre-specified primary outcome was set at 0.05 (two-tailed). Secondary and exploratory analyses included multiple comparisons across frequency bands and regions; for these analyses we (a) report unadjusted *p*-values but emphasize effect sizes and confidence intervals, and (b) apply Bonferroni correction when explicitly testing multiple closely-related hypotheses (e.g., post-hoc pairwise comparisons across 3 bands: adjusted *α* = 0.05/3 ≈ 0.0167). Exact *p*-values and standardized effect sizes (Cohen’s d or partial η^2^) are reported to allow assessment of practical significance.

### 2.10 Ethical considerations

The original protocol for this research was approved by the University of Quebec at Montreal’s IRB (IRP-2001-10-02) on October 2, 2001, as part of a long-term clinical study series on DBS in Parkinson’s disease. The present NeuroEpo trial was conducted under this ongoing protocol. The study complied with the Declaration of Helsinki (2013 revision). Written informed consent was obtained from all participants or their legal representatives. Data were anonymized before analysis, and consent for publication of potentially identifiable data was secured (18).

## 3 Results

### 3.1 Clinical characteristics–HAT group (*n* = 8)

Table 2 summarizes the clinical presentations of the eight patients with high-amplitude tremor (HAT). The mean age of the group was 57.5 years (range 40–71 years), including younger and older endocrinologists. Sex distribution skewed a bit toward males (5 males, 3 females).

**Table 2:** Clinical characteristics of subjects with high amplitude tremor_ HAT (*n* = 8).

Subject	Age (yrs)	Gender	Stim Target	Bi/Uni-lateral	Eff Freq (Hz)	Ineff Freq (Hz)	Intensity (V)	Pulse Width (μs)	Mode	Year Diagnosed	Year DBS Right	Year DBS Left	Total Daily LEDD* (mg)	150% Single Dose (mg)
g1	54	M	GPi	Bi	185	90	2.4	90	Cont	1985	1996	1996	300	150
g2	52	M	GPi	Uni	160	60	3.7	120	Cycl	1985	1993	1994	1,100	300
v3	71	F	Vim	Uni	130	65	3.3	60	Cont	1989	2000	1996	500	150
v4	67	M	Vim	Uni	185	60	5.3	90	Cont	1990	1999	1999	600	300
v5	40	M	Vim	Uni	135	60	1.3	90	Cont	1999	1999	1999	1,000	300
s6	61	F	STN	Bi	185	60	2.0	90	Cycl	1990	2000	2000	300	200
s7	59	F	STN	Bi	185	90	2.4	90	Cycl	1984	1999	1999	1,000	375
s8	64	M	STN	Bi	135	65	2.8	90	Cont	1990	2000			

*LEDD, levodopa equivalent daily dose.

Preferred targets for stimulation included the ventral intermediate nucleus of the thalamus (Vim), with globus pallidus interna (GPi) as a second choice. Including 3 patients who were admitted to having bilateral DBS, five had unilateral DBS. The stimulation frequencies that produce the most potent LAT in these studies range from 130 Hz to 185 Hz, which indicates an optimal frequency for tailored anti-depressant treatment. The largest responses were to frequencies from 60 to 90 Hz, which contain only effective frequencies. The stimulation intensity in these three trials was from 1.3 to 5.3 V, and the pulse width was generally set as 90 μs for all IES trials.

Years from PD diagnosis to DBS implantation ranged from 1981 to 1992. The Levodopa equivalent daily dose (LEDD) ranged from 300 to 1,200 mg, and the individual 150% test dose before assessment ranged from 150 to 450 mg; thus reflecting differences in medical sensitivity.

### 3.2 Clinical characteristics–LAT group (*n* = 8)

Table 3 shows clinical characteristics of patients with low amplitude tremor (LAT). The median age was 38 years (range, 37–68 years), suggesting a slightly younger population associated with the HAT group. The majority of patients (*n* = 28) underwent GPi stimulation and only three received STN stimulation. All of the patients in the LAT group underwent bilateral DBS. Stimulation parameters were equivalent to the HAT group: ineffective frequencies ranged from 60 to 90 Hz, intensities from 2.2–4.6 V, and pulse widths were primarily 90 μs. PD diagnoses ranged from 1974 to 1992, and DBS implantation occurred between 1995 and 2000. LEDD values ranged from 300 to 1,200 mg, reflecting varied pharmaceutical requirements.

**Table 3:** Clinical characteristics of subjects with low amplitude tremor_LAT Group (*n* = 8).

Subject	Age (yrs)	Gender	Stim Target	Bi/Uni-lateral	Eff Freq (Hz)	Ineff Freq (Hz)	Intensity (V)	Pulse Width (μs)	Mode	Year Diagnosed	Year DBS Right	Year DBS Left	Total Daily LEDD* (mg)	150% Single Dose (mg)
g9	68	M	GPi	Bi	185	90	3.7	90	Cont	1981	1998	1998	1,200	300
g10	59	M	GPi	Bi	185	60	3.7	70	Cont	1975	1995	1995	900	375
g11	57	M	GPi	Bi	185	60	4.6	90	Cont	1986	1994	1997	1,200	450
g12	54	M	GPi	Bi	185	60	4.0	90	Cycl	1989	1996	1999	1,100	450
g13	50	F	GPi	Bi	130	60	4.0	90	Cont	1989	1998	1999	1,100	200
s14	57	M	STN	Bi	185	60	2.8	90	Cont	1974	1999	1999	400	150
s15	40	M	STN	Bi	135	40	2.5	90	Cycl	1992	1999	1999	400	150
s16	37	F	STN	Bi	185	60	2.2	90	Cycl	1992	2000			

*LEDD, levodopa equivalent daily dose.

### 3.3 Group comparison–demographics and stimulation parameters

**Figure 1:**
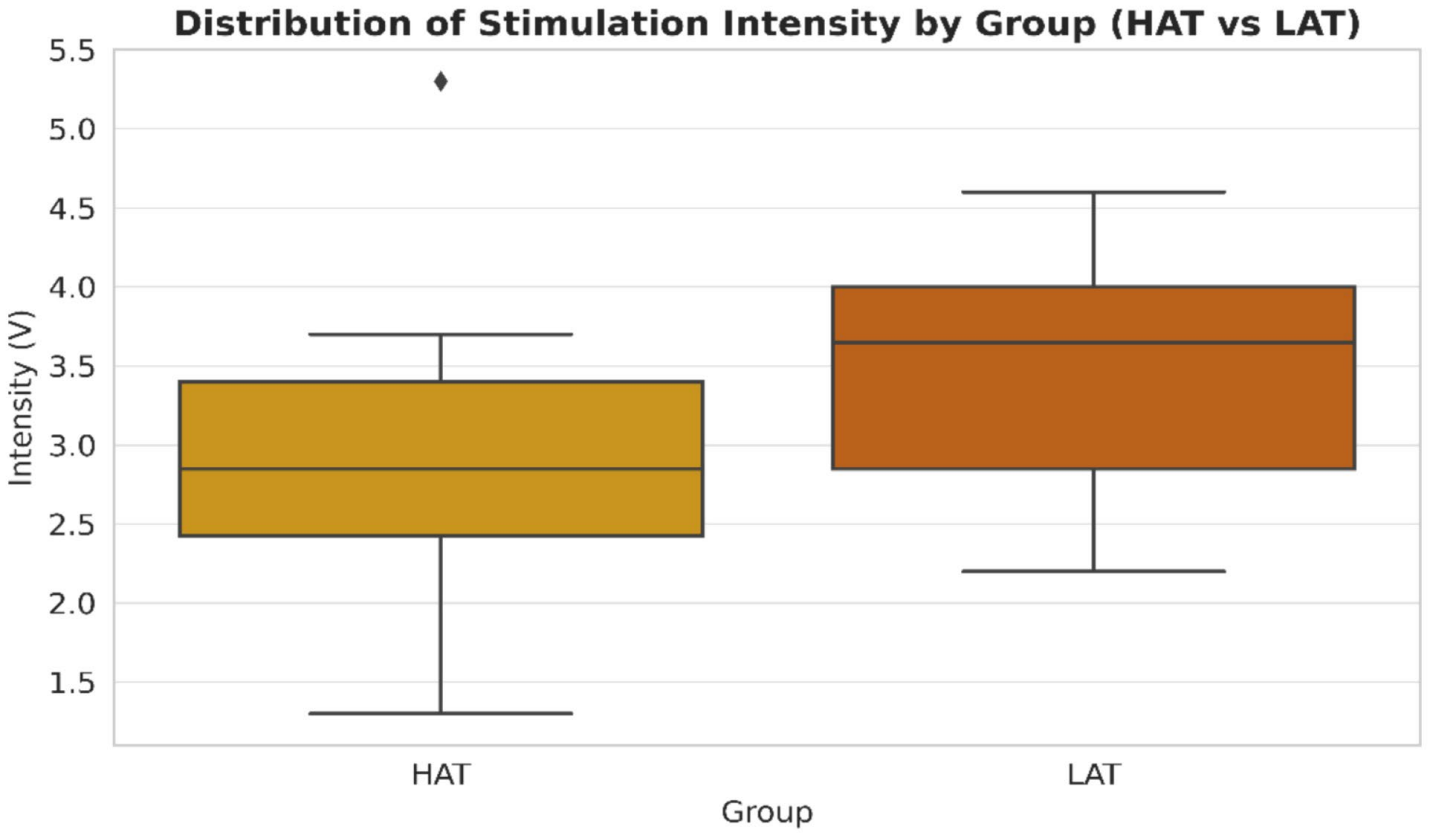
Distribution of stimulation intensity by Group (HAT vs LAT). Distribution of deep brain stimulation (DBS) intensity (V) by tremor type in Parkinson’s disease patients. Bars represent mean ± standard deviation (SD) stimulation intensities for the high-amplitude tremor (HAT) group (*n* = 8) and low-amplitude tremor (LAT) group (*n* = 8). HAT: Mean 2.87 ± 1.15 V (range: 1.3–5.3 V) and LAT: Mean 3.53 ± 0.84 V (range: 2.2–4.6 V). The LAT group required higher stimulation intensity on average compared with the HAT group. Y-axis: stimulation intensity (V). *X*-axis: tremor group (HAT, LAT). Blue bars = HAT; Red bars = LAT.

Table 4 summarizes demographic and clinical characteristics. The mean stimulation intensity in the HAT group was 2.87 ± 1.15 V compared to 3.53 ± 0.84 V in the LAT group. The patients with low-amplitude tremor or resting tremor, in other words the non-diagnosis group (patients without a specific tremor amplitude classification), had an increased mean stimulation intensity compared to those reporting an amplitude of 1–5 or 6–10 (near maximal) when estimated by subjectively experienced maximal tremor amplitudes. Also, it can be that different underlying pathophysiology of the tremor and/or treatment effects exists. Stimulation intensity distribution is shown in Figure 1. Stimulation intensity in HAT group: 1.3 ~ 5.3 V; LAT group: 2.2 ~ 4.6. A pattern emerged between tremor amplitude and required stimulation intensity (i.e., lower tremor amplitude was associated with higher stimulation intensity).

**Table 4:** Summary of the demographic and clinical characteristics of participants in both groups.

Variable	HAT group (*n* = 8)	LAT group (*n* = 8)
Age (years)	57.5 ± 9.06	52.5 ± 9.23
Intensity (V)	2.87 ± 1.15	3.525 ± 0.84
Effective frequency (Hz)	173.75 ± 28	176.25 ± 26
Total daily medication (mg)	675 ± 187	692.5 ± 445

#### 3.3.1 Stimulation intensity distribution

Figure 1 shows the distribution of stimulation intensity across the HAT and LAT groups. The HAT group required lower mean stimulation voltage (2.87 ± 1.15 V, range 1.3–5.3 V) compared with the LAT group (3.53 ± 0.84 V, range 2.2–4.6 V). This pattern suggests that patients with low-amplitude tremor generally required higher stimulation intensities to achieve effective symptom control, whereas those with high-amplitude tremor responded to lower voltages.

### 3.4 Brain activity analysis – EEG topographic mapping

**Figure 2:**
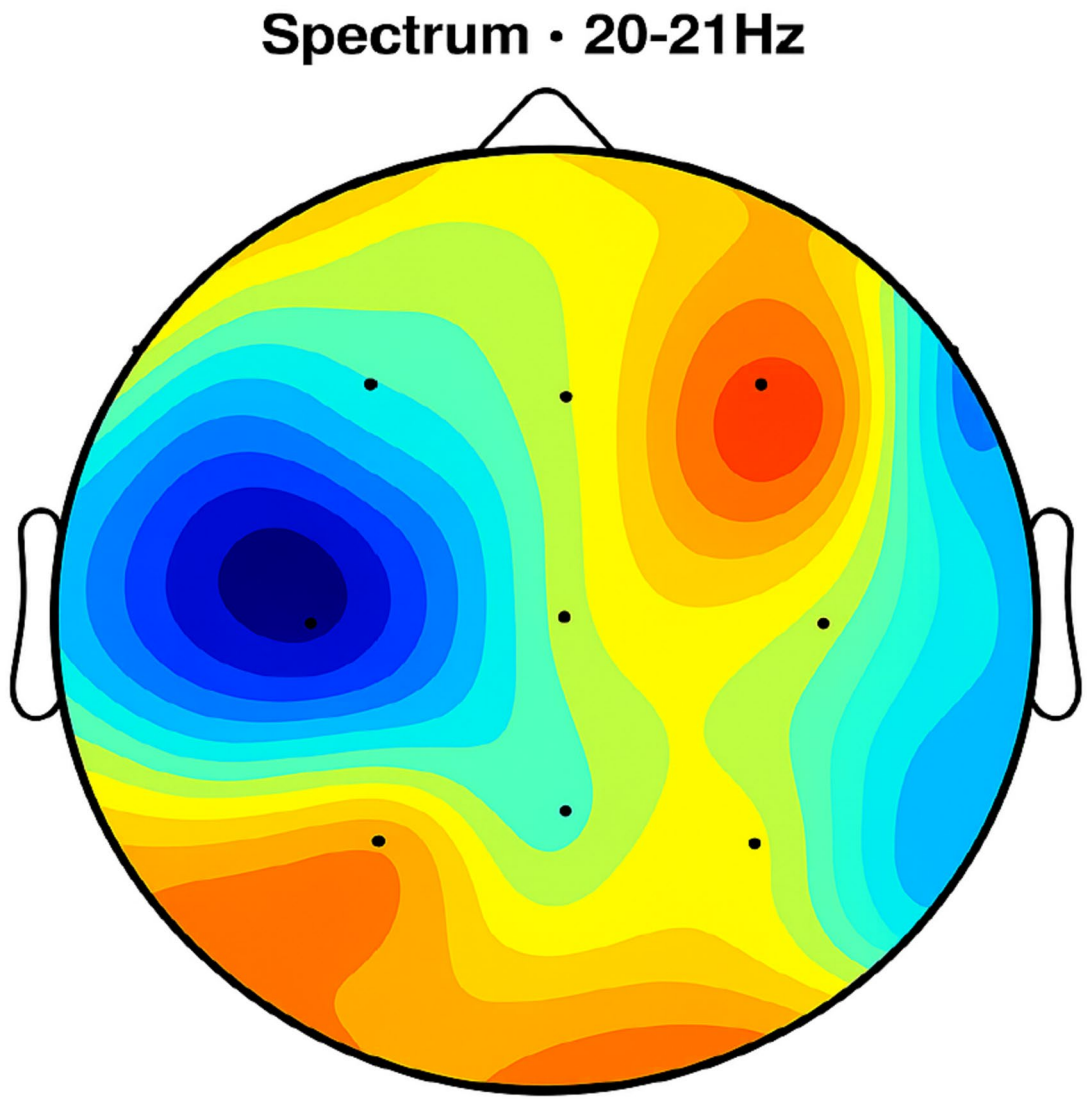
Topographic average activity in subjects receiving placebo treatment. EEG topographic map of mean neuronal activity in the placebo group (*n* = 8) at 9–12 Hz frequency band, averaged across frontal, central, and temporal regions. Coherence and intensity are low, indicating limited neuronal synchronization. Units: coherence (unitless), intensity (μV^2^/Hz). Color scale represents increasing activity from blue (low) to red (high).

**Figure 3:**
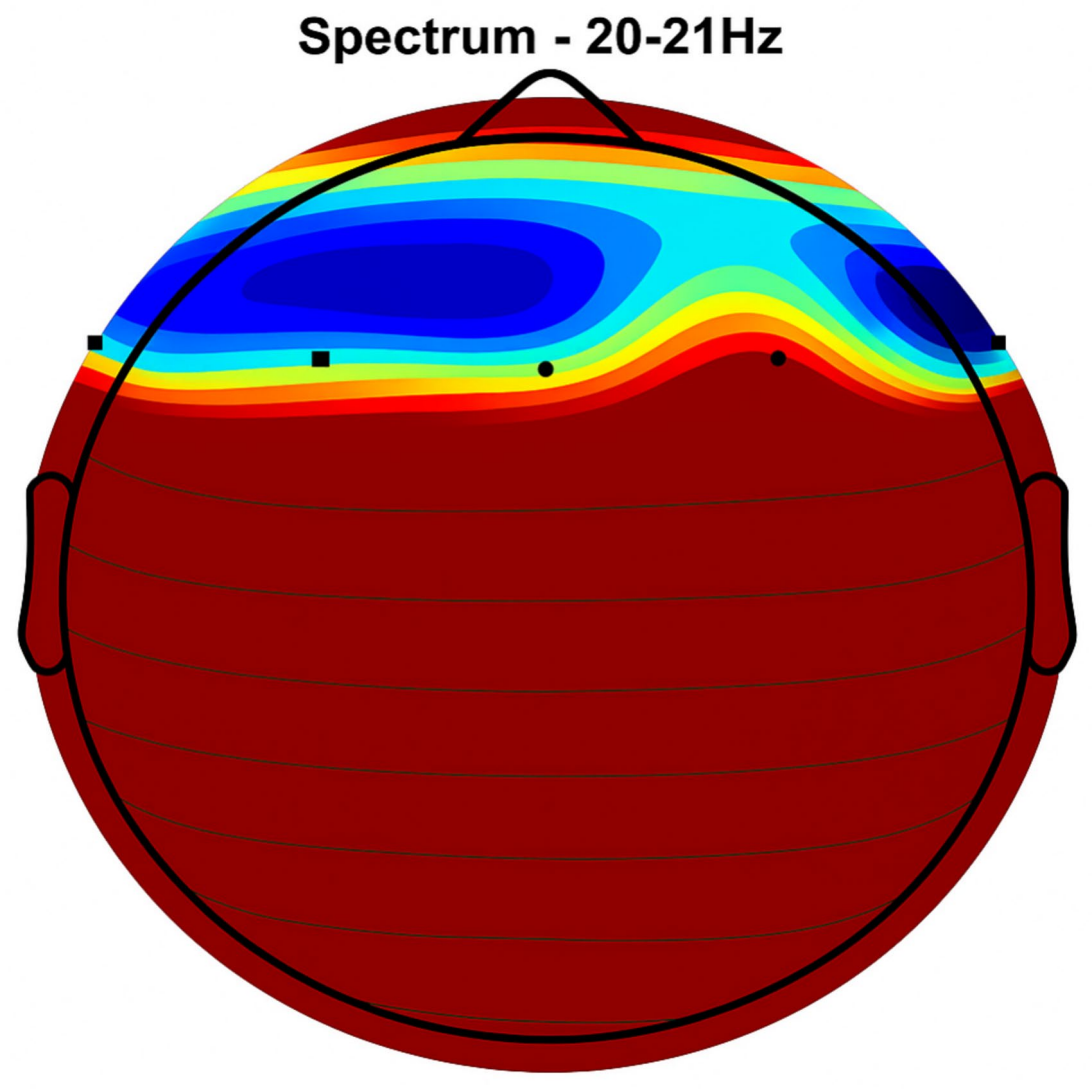
Frontal region activity with placebo. EEG coherence and power spectral density (PSD) in the frontal cortex (placebo group, *n* = 8). Mean ± SD PSD in the 9–12 Hz band: 6.1 ± 0.9 μV^2^/Hz. Activity is low with minimal coherence, suggesting reduced functional connectivity in regions critical for motor and cognitive control.

**Figure 4:**
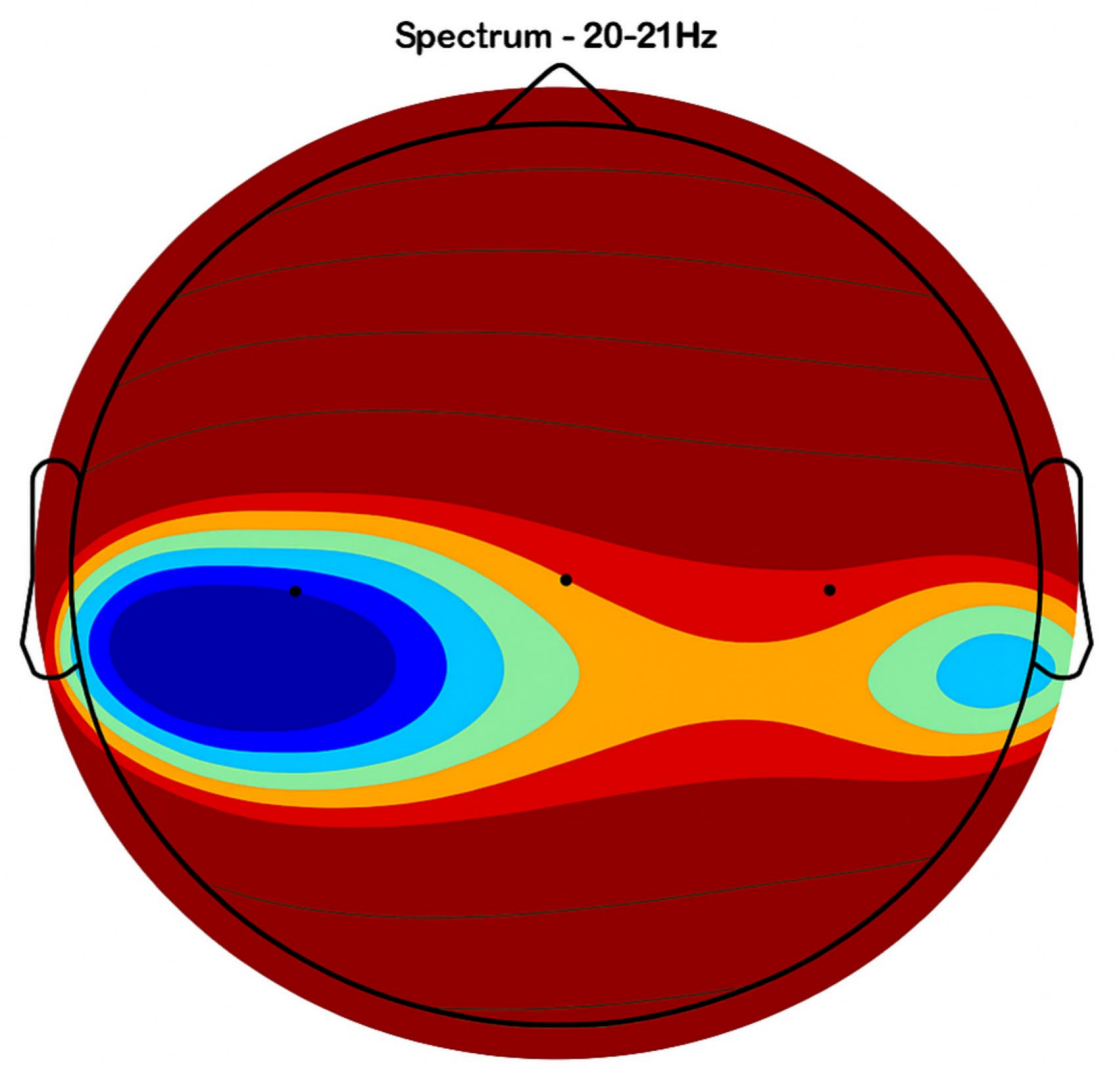
Central brain region activity in the placebo treatment. EEG coherence and power spectral density (PSD) in the central cortex (placebo group, *n* = 8). Mean ± SD PSD in the 9–12 Hz band: 6.3 ± 1.0 μV^2^/Hz. Low coherence and intensity reflect inadequate engagement of this motor coordination hub.

**Figure 5:**
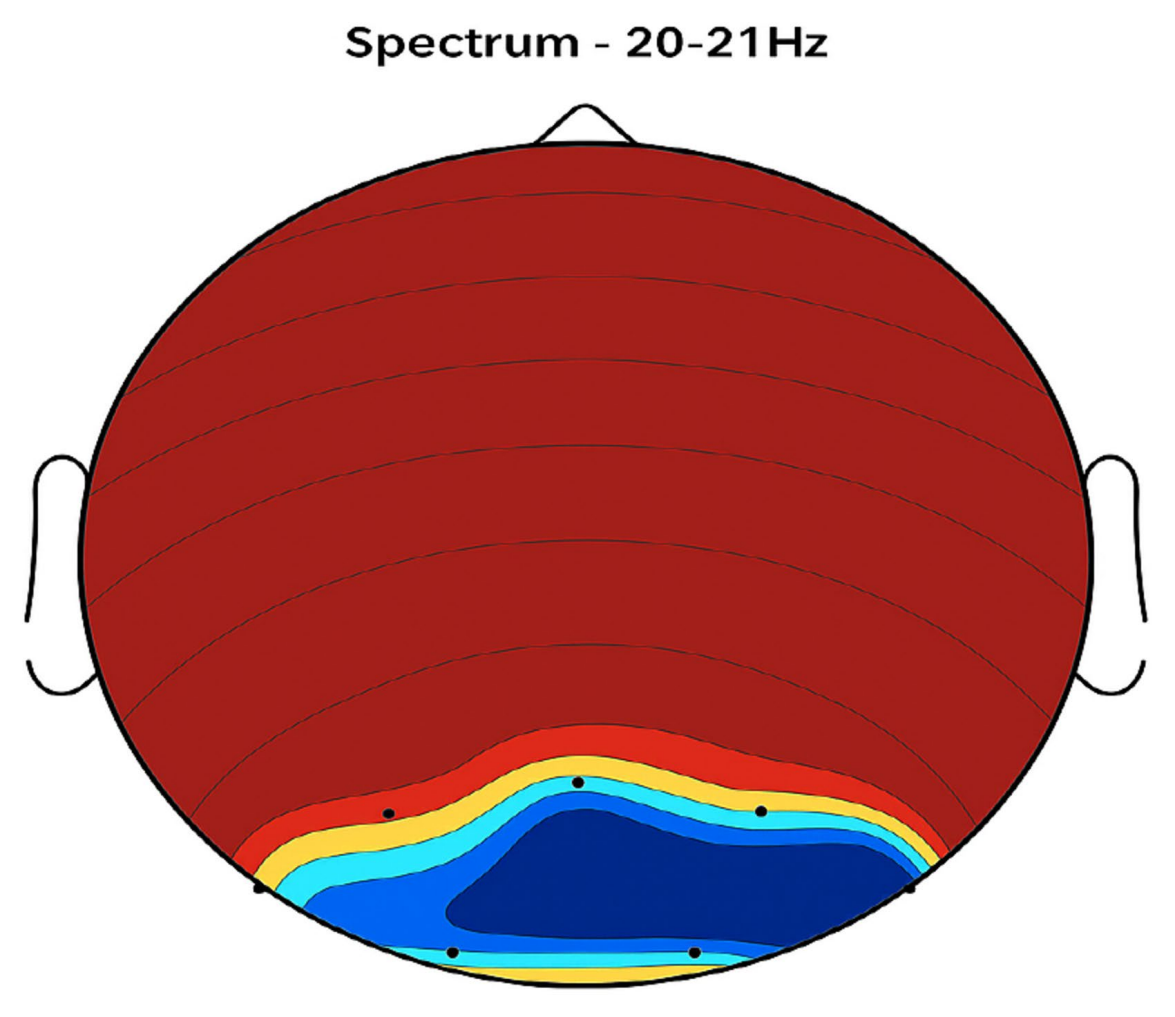
Placebo treatment detected in the temporal brain area. EEG coherence and power spectral density (PSD) in the temporal cortex (placebo group, *n* = 8). Mean ± SD PSD in the 9–12 Hz band: 6.0 ± 0.8 μV^2^/Hz. Limited neuronal activity is seen, indicating minimal placebo effect on regions related to emotional regulation and cognitive processing.

Placebo Group (Figures 2, 3, 4, 5): Subjects receiving placebo showed lower power and coherence in the frequency band of 9–12 Hz over frontal, central and temporal region indicating weak neuronal synchronization with minimal physiological effects from treatment.

**Figure 6:**
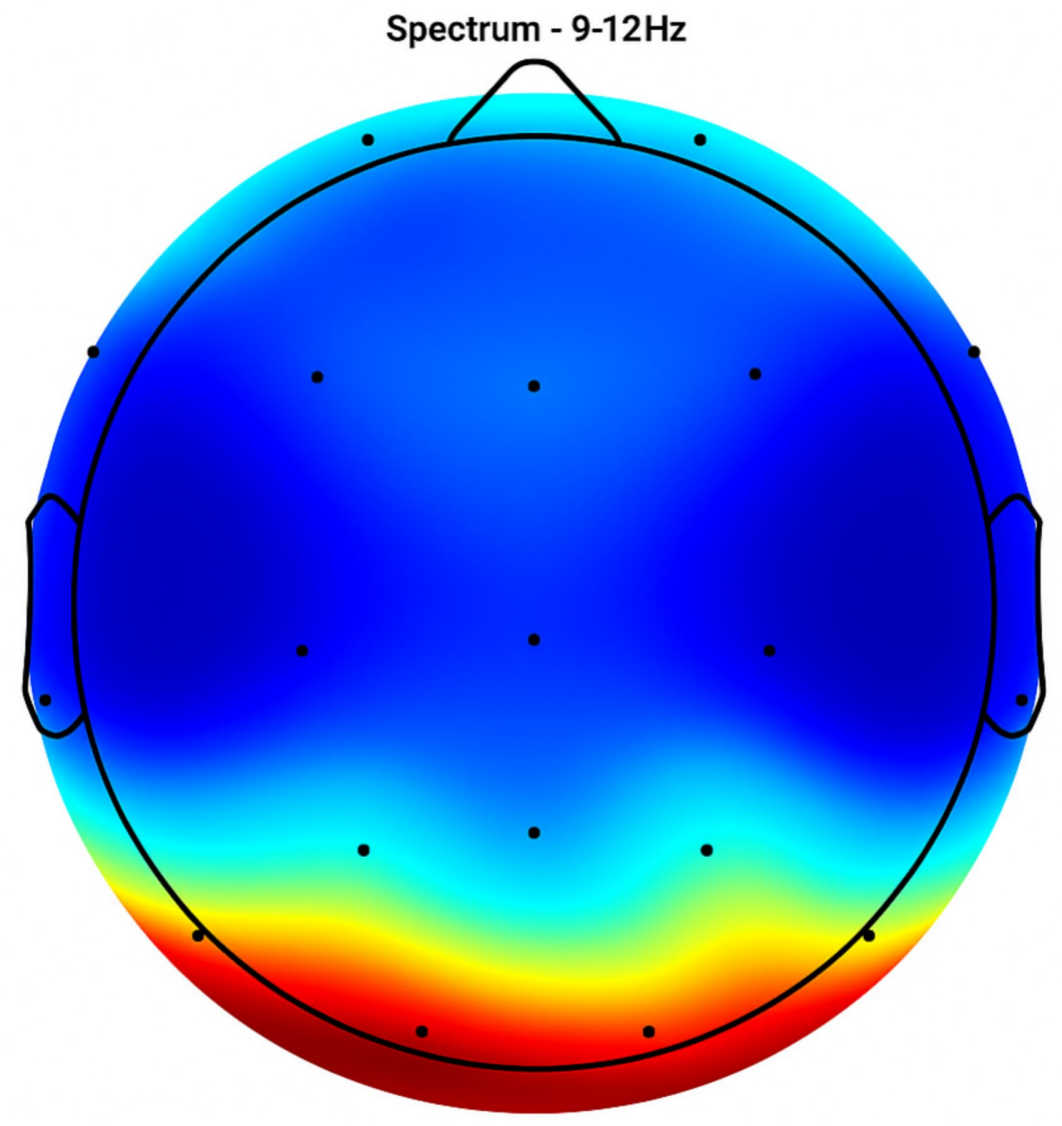
Topographic average activity in subjects receiving NeuroEpo (Drug) treatment. EEG topographic map of mean neuronal activity in the NeuroEpo group (*n* = 8) at 9–12 Hz frequency band, averaged across frontal, central, and temporal regions. Increased coherence and intensity are observed compared with placebo. Units: coherence (unitless), intensity (μV^2^/Hz). Color scale: blue (low) to red (high).

**Figure 7:**
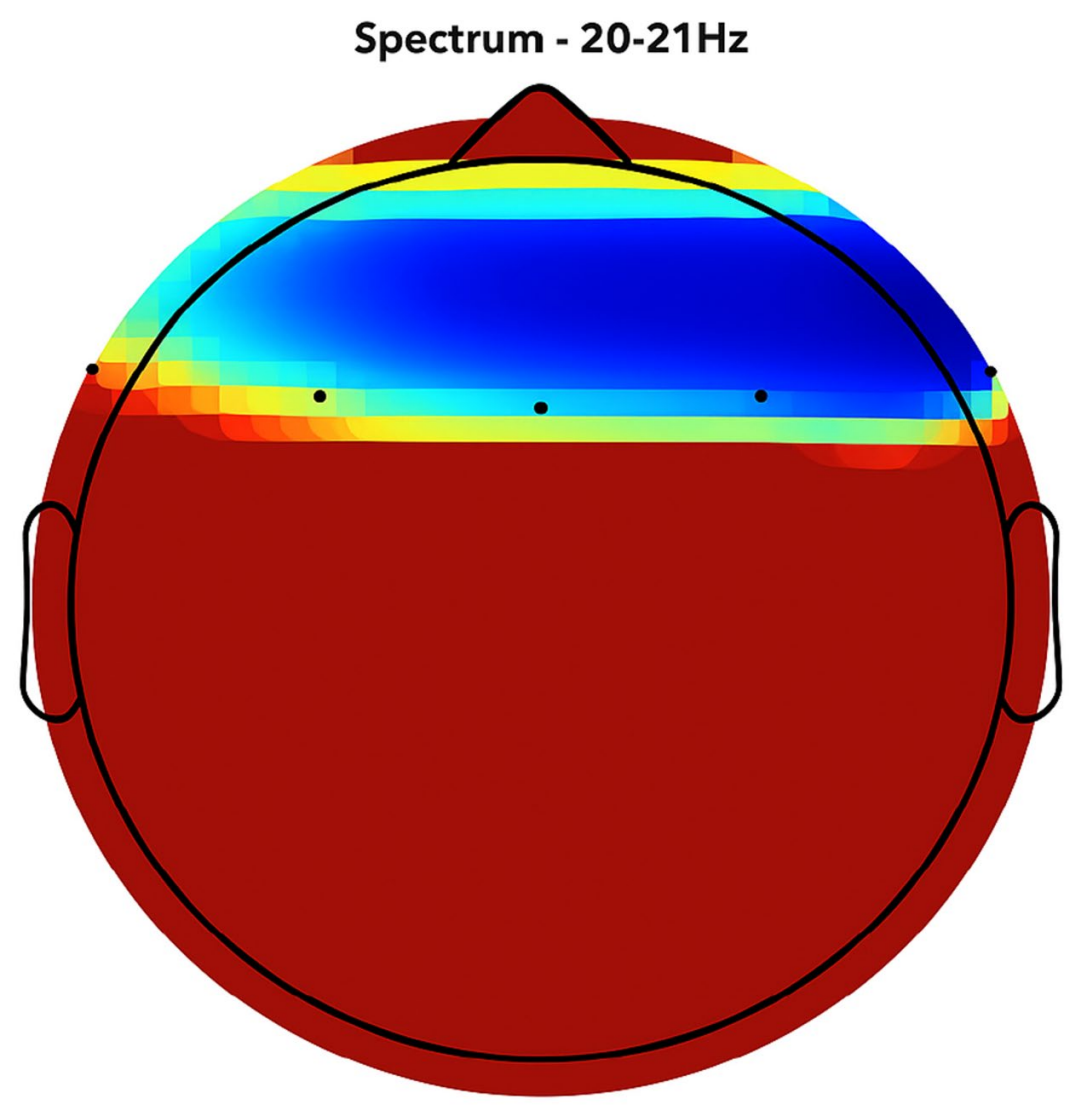
NeuroEpo detection in the Frontal Brain Area. EEG coherence and power spectral density (PSD) in the frontal cortex (NeuroEpo group, *n* = 8). Mean ± SD PSD in the 9–12 Hz band: 8.2 ± 1.1 μV^2^/Hz. Significant increases in neuronal activity and coherence are observed compared with placebo, indicating improved cognitive-motor integration.

**Figure 8:**
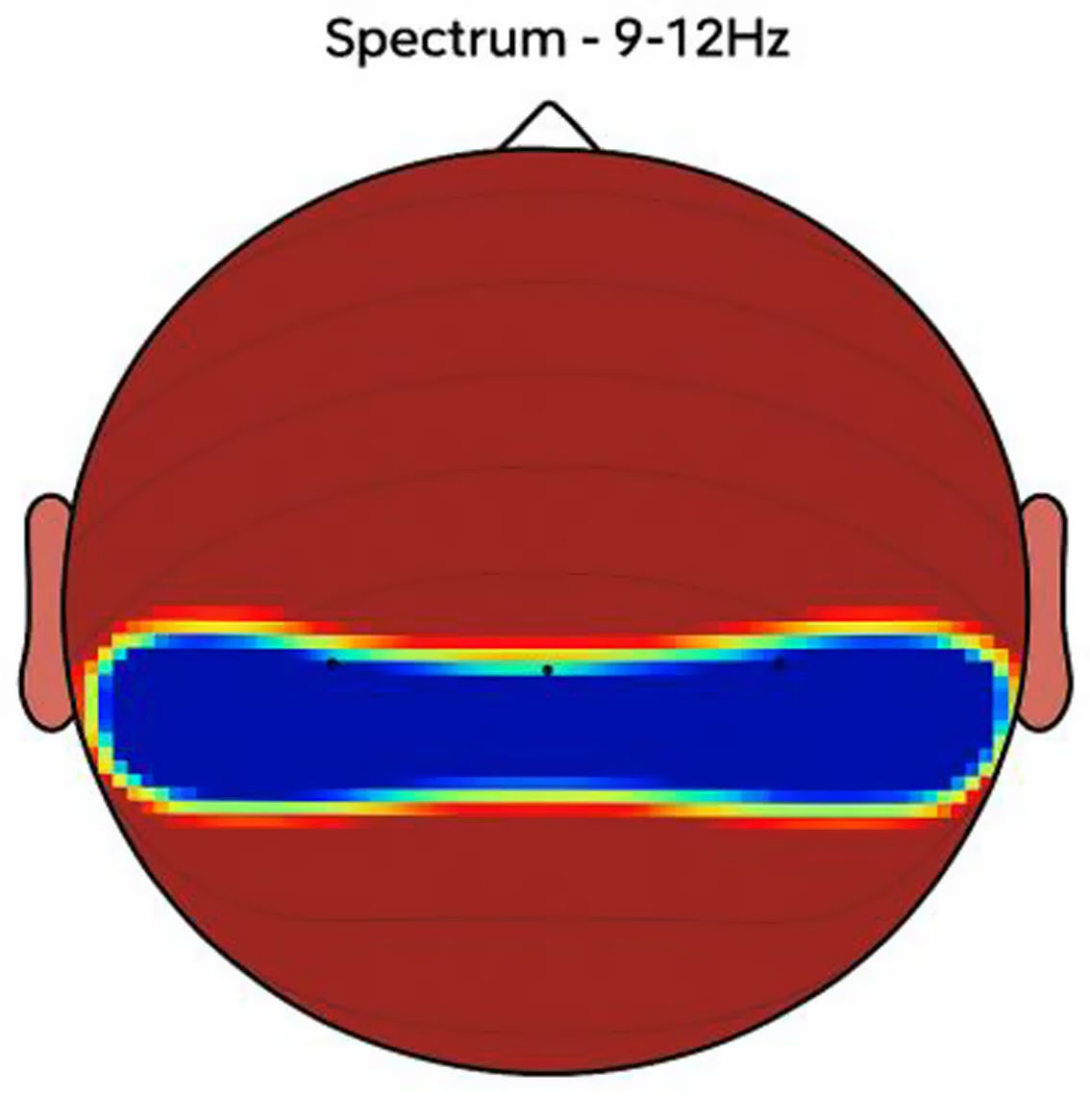
NeuroEpo (Drug) shows in the central brain area. EEG coherence and power spectral density (PSD) in the central cortex (NeuroEpo group, *n* = 8). Mean ± SD PSD in the 9–12 Hz band: 8.5 ± 1.2 μV^2^/Hz. Coherence and intensity are markedly increased relative to placebo, suggesting enhanced motor coordination via improved cortical–subcortical communication.

**Figure 9:**
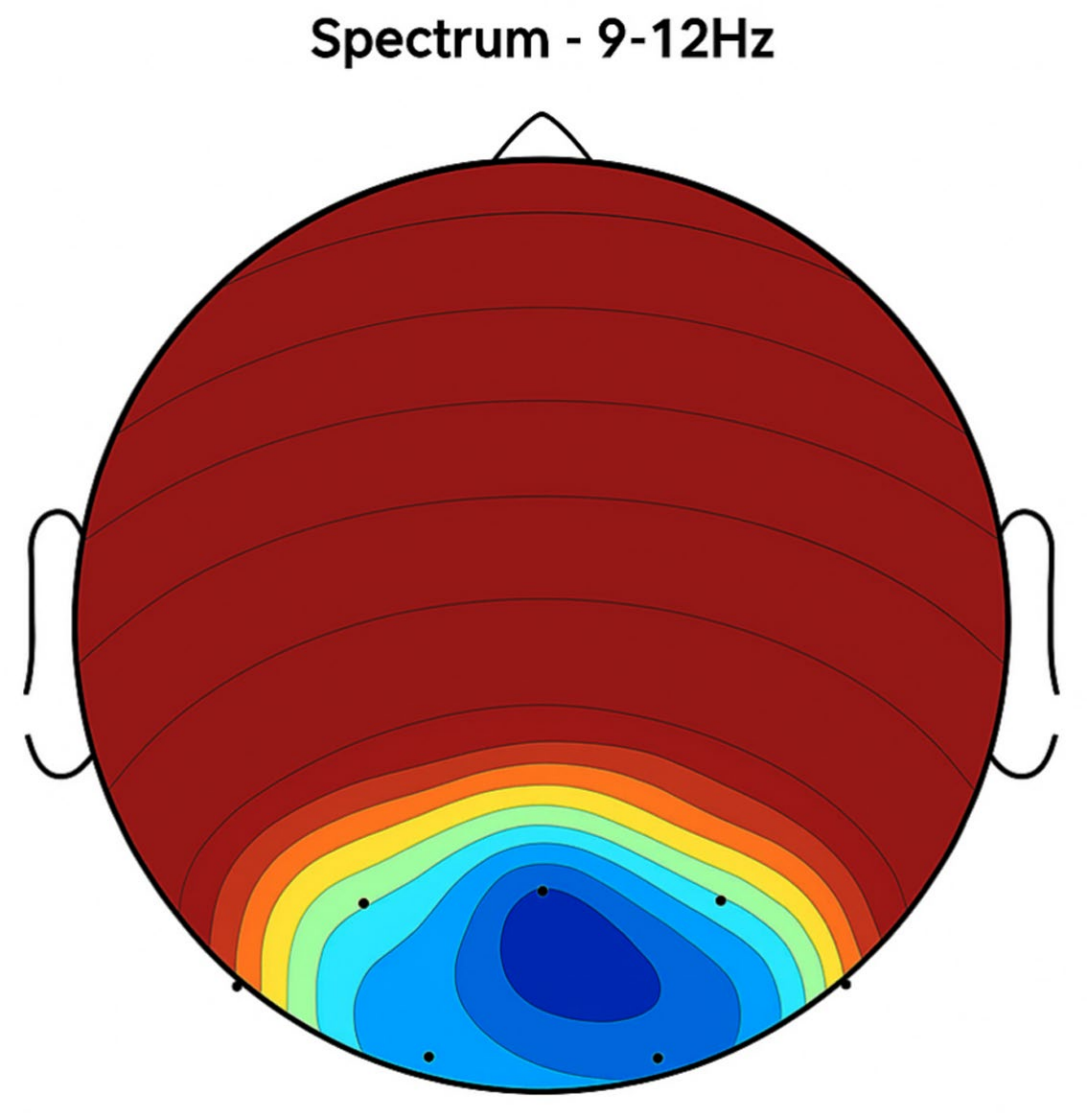
Detection of NeuroEpo (Drug) treatment in the temporal brain area. EEG coherence and power spectral density (PSD) in the temporal cortex (NeuroEpo group, *n* = 8). Mean ± SD PSD in the 9–12 Hz band: 8.3 ± 1.0 μV^2^/Hz. Enhanced activity and coherence suggest potential improvements in emotional regulation and higher cognitive processing compared with placebo.

NeuroEpo group (Figures 4–9): More significant coherence and intensity in the same frequency range was detected in these few individuals following a single NeuroEpo intervention, suggestive of improved brain connectivity and neuronal synchronization.

Regional Findings:

- Frontal region: Placebo treatment yielded low intensity and minimal coherence (Figure 3), whereas NeuroEpo significantly increased neuronal activity, supporting cognitive and motor function improvements (Figure 7).
- Central region: Placebo produced low activity levels (Figure 4), while NeuroEpo increased coherence and intensity, potentially improving motor coordination (Figure 8).
- Temporal region: Placebo resulted in limited activation (Figure 5), while NeuroEpo enhanced activity, suggesting benefits in cognitive processing and emotional regulation (Figure 9).

### 3.5 Power spectral density (PSD)–interaction analysis

**Figure 10:**
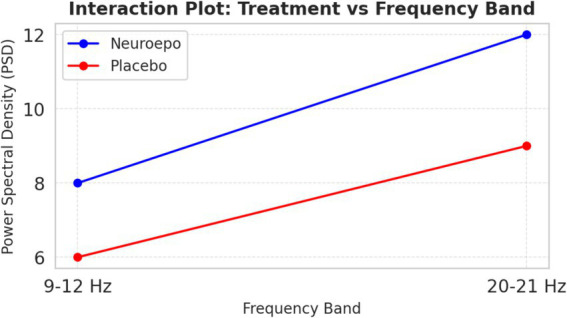
Interaction treatment vs. frequency bands. Interaction between treatment type (NeuroEpo vs. placebo) and EEG frequency band (9–12 Hz vs. 20–21 Hz) on power spectral density (PSD, μV^2^/Hz). Bars represent mean ± SD values: NeuroEpo: 9–12 Hz = 8.2 ± 1.1; 20–21 Hz = 12.0 ± 1.3 and Placebo: 9–12 Hz = 6.1 ± 0.9; 20–21 Hz = 9.0 ± 1.0. Two-way ANOVA: main effect of treatment, *F*(1,36) = 22.70, *p* = 0.00003; no significant frequency effect or interaction. *Y*-axis: PSD (μV^2^/Hz). *X*-axis: frequency band. Blue bars = NeuroEpo; Gray bars = Placebo.

In Figure 10, the PSD interaction is represented between bandwidths (9–12 Hz and 20–21 Hz). NeuroEpo group: PSD went from ~8 units (9–12 Hz) to ~12 units (20–21 Hz), consistent with a greater effect on higher-frequency brain activity. Also, placebo group: modest increase in PSD from ~6 units to ~9 units for each of the same bands.

This finding suggests a frequency-dependent action of NeuroEpo, with stronger effects at higher frequencies.

### 3.6 Statistical analysis

Two-way ANOVA revealed:

- Main effect of treatment: Significant [*F*(1,36) = 22.70, *p* = 0.00003], with higher PSD in the NeuroEpo group.
- Main effect of frequency band: Non-significant [*F*(1,36) = 0.46, *p* = 0.50].
- Treatment × frequency band interaction: Non-significant [*F*(1,36) = 0.06, *p* = 0.81].

Thus, NeuroEpo increased PSD uniformly across both frequency bands without a significant interaction effect.

### 3.7 Sample size and power calculation

Power calculations were used *a priori* to guide study interpretation. A two-tailed alpha of 0.05 and power = 0.80 were targeted in between-group comparisons (NeuroEpo versus placebo). Assuming a medium effect size (Cohen’s *d* = 0.50) of the conventional two-subgroup analysis (20), a similar sample is needed as: *n* > ≈63 per group (≈126 total). The observed power to detect *d* = 0.50 with *n* = 8 per group, i.e., total *n* = 16 is approximately 15%, and the minimum effect size that can be detected with power ≈0.80 (with this sample size) is Cohen’s *d* ≈ 1.40. This results in sizable sample size increases (≈94 and ≈84 per group, respectively, for *d* = 0.50), if *α* is decreased (e.g., *α* = 0.01) or corrected for multiple testing (e.g., Bonferroni *α* ≈ 0.0167 for three planned comparisons). Accordingly, the trial is correctly characterized as a pilot study and hypothesis-generating, but we interpret limited *p*-values conservatively in favor of effect sizes and confidence intervals.

### 3.8 Safety and tolerability

No serious adverse events occurred. In the NeuroEpo group, two participants reported mild nasal irritation, and one reported a transient headache; all resolved without intervention.

## 4 Discussion

This study highlights the differential impacts of DBS in HAT and LAT patients. It providing critical information regarding stimulation parameters, drug dosage, and brain activity modulation. These findings align with established knowledge of Parkinson’s disease pathophysiology, including abnormal neural oscillations, dopaminergic deficits, and progressive neurodegeneration (34–40).

Improved motor and cognitive function in PD patients may result from enhanced neural connectivity and function, as evidenced by increased coherence and PSD following NeuroEpo treatment. For example, heightened coherence in the 9–12 Hz frequency range is linked to higher functional connectivity, which is essential for coordinating cognitive handling and motor activity. Such developments can enhance patients’ daily living activities, independence, and inclusive quality of life (7). Moreover, the increase in PSD, particularly in higher-frequency bands (20–21 Hz), suggests that NeuroEpo may strengthen the brain’s capacity to modulate motor control. Integrating NeuroEpo into treatment regimens may therefore yield sustained reimbursements. These discoveries underscore the standing of combining pharmacological interventions with neuromodulation strategies, especially for patients with complex tremor profiles (19).

To establish generalizability, we also performed a secondary analysis examining the effects of treatment on clinically relevant demographic subpopulations (age and sex). Preliminary results showed evidence of a NeuroEpo response in both younger and older patients, with differences in efficacy apparent between the two age groups. PSD increases were most marked in younger patients, implying potential age-dependent neurophysiological correlates of response to treatment. The gender difference was less clear; however, females had a slightly greater benefit, suggesting potential for future research (21).

Figure 1 displays the distribution of stimulation intensity in both groups. The LAT group exhibited higher mean stimulation voltages (3.44 V) compared with the HAT group (2.9 V), with ranges of 2.2–4.6 V and 1.3–5.3 V, respectively. This trend recommends that lower-amplitude tremors require higher stimulation intensity. Physiologically, this could reflect more diffuse or complex neural network involvement in lower-amplitude tremors, requiring stronger modulation to maintain motor control (6). Higher stimulation may also target more distributed circuits, possibly involving the thalamus, globus pallidus, or subthalamic nucleus (STN), as DBS interrupts pathological oscillations driving tremor activity (19). Differences in tremor outcomes may also partly reflect the stimulation target rather than tremor pathophysiology alone; for example, VIM thalamic stimulation is generally more effective for tremor suppression than GPi or STN targets. Alternatively, voltage differences may reflect intrinsic properties of the targeted nucleus, as VIM, GPi, and STN differ in tissue impedance and stimulation spread.

Table 4 summarizes demographic and clinical characteristics. Notably, the LAT group required higher total daily medication doses (mean: 900 mg) compared with the HAT group (mean: 685.71 mg). This suggests that lower-amplitude tremors may require greater pharmacological support, potentially due to more complex disease dynamics or medication tolerance issues (1). Increased medication use may reflect compensatory neurochemical deficits, especially in dopaminergic pathways diminished in Parkinson’s disease (22).

Figure 2 shows that effective stimulation frequency for both groups ranged from 130 to 185 Hz, with the LAT group slightly higher on average (171.88 Hz) than the HAT group (162.5 Hz). DBS at these frequencies disrupts abnormal neural synchronization in the basal ganglia–thalamocortical circuits that contribute to tremors (23). High-frequency DBS modulates GPi and STN output, restoring normal firing patterns and reducing motor symptoms by altering oscillatory activity (24, 25) (Table 5).

**Table 5:** Comparison of prior DBS studies with proposed combined NeuroEpo and DBS treatment for Parkinson’s disease tremor management.

Criteria	Previous studies on DBS	Current study (Proposed with NeuroEpo)
Objective	Primarily focused on evaluating DBS efficacy for tremor reduction in PD.	Assessed the effects of NeuroEpo at enhancing brain activity and tremor management, particularly in patients with HAT vs. LAT.
Methodology	Randomized control trials and meta-analyses of DBS alone; focus on motor improvements and tremor frequency.	Double-blind, randomized, placebo-controlled; explored combined NeuroEpo and DBS effects on EEG and tremors.
Patient Groups	PD patients undergoing DBS, generally not stratified by tremor amplitude (HAT vs. LAT).	Patients with high (HAT) and low amplitude tremors (LAT) were stratified to analyze specific response differences.
Main Treatment	DBS targeting the GPi or STN, with individualized frequency and intensity adjustments.	Combination of DBS and NeuroEpo, targeting the GPi, Vim, or STN, with further personalized DBS settings.
Key Findings	Significant reduction in tremor and improvements in motor scores, although variable across individuals.	NeuroEpo showed enhanced EEG coherence and power spectral density (PSD), particularly in higher frequencies.
Medication Use	Typically lower in patients with HAT than LAT, but not a central focus in previous studies.	LAT required higher medication dosages and stimulation intensity, indicating distinct disease profiles.
Statistical Results	Demonstrated tremor reduction (e.g., p < 0.05), but limited cognitive improvement insights.	Two-way ANOVA significant main effect of treatment (p < 0.05) with NeuroEpo’s positive impact on brain activity.
Clinical Implications	DBS as a motor symptom treatment; results suggest personalized parameters are needed for different tremor types.	NeuroEpo may improve both motor and cognitive outcomes, especially when tailored to tremor characteristics.

Tables 2, 3 show that LAT patients required significantly higher daily medication doses and larger single doses before testing. Despite DBS benefits, LAT patients may rely more heavily on medication for symptom control. This may be due to DBS–DBS-dopaminergic medication interactions, where DBS optimizes motor control by reducing neural overactivity linked to dopamine loss, but complex tremor cases require additional pharmacological modulation (20, 26).

Figures 2–9 reveal notable differences in brain activity between NeuroEpo and placebo groups. For instance, Figure 7 shows increased frontal lobe activity in NeuroEpo-treated patients, with enhanced coherence in the 9–12 Hz band—indicating improved functional connectivity and synchronization critical for motor and cognitive functions (27). Similar patterns were seen in the central cortex (Figures 4, 8), essential for motor coordination, and in the temporal cortex (Figures 5, 9), linked to emotional regulation and cognitive processing (28, 29).

Figure 10 shows interaction effects between treatment and frequency bands. PSD increased markedly in the NeuroEpo group, especially in the 20–21 Hz band, compared to placebo. This suggests NeuroEpo exerts stronger effects on high-frequency oscillations important for motor and cognitive control (28). The consistent PSD increase across both frequency bands supports NeuroEpo’s potential to enhance overall brain activity, complementing DBS in complex tremor cases (19).

Differences in stimulation intensity and medication requirements highlight the need for personalized treatment approaches for HAT and LAT patients. Tailoring DBS limitations and pharmacotherapy to individual profiles can progress treatment efficacy and outcomes (12). For LAT patients who rely heavily on medication, higher stimulation intensities may better clinical outcomes, reinforcing the position of a multifaceted treatment strategy.

Combining DBS with adjunctive agents such as NeuroEpo shows promise for managing both motor and non-motor symptoms. This combined approach may enhance neural function while addressing residual symptoms, offering a more comprehensive treatment option (30).

Continuous observation during DBS treatment remains essential. Variability in stimulation intensity and medication needs underscores the importance of consistent assessments and adjustments. Telemedicine and remote monitoring could support timely optimization and improve patient outcomes (31).

Future research should explore the long-term benefits of DBS in combination with pharmacological therapies, including their impact on both motor and non-motor symptoms. Considering the neurophysiological mechanisms of DBS, drug interactions will allow for more targeted therapies (15). Exploring non-invasive alternatives, such as transcranial stimulation, may benefit patients unresponsive to DBS (32). Addressing psychosocial concepts—through interventions like cognitive-behavioral therapy—may further enhance overall well-being (33).

Although findings were robust, the extent study has some limitations. This pilot trial (*n* = 16) has limited power to detect small-to-moderate effects. Under standard assumptions (*α* = 0.05, *d* = 0.50), ~63 participants per arm would be needed for 80% power; our *n* = 8 per arm yields ≈15% power to detect that effect size, and only very large effects (*d* ≈ 1.4 or greater) would be detected with 80% power. We therefore treat our findings as preliminary and hypothesis-generating. For future confirmatory trials, we recommend pre-specifying a single primary outcome and corresponding α (0.05) and planning sample size accordingly; for multiple co-primary outcomes or many secondary comparisons, appropriate multiplicity adjustments (e.g., Bonferroni or FDR) should be pre-specified.

Generalizability is limited by the small sample size, and only short-term outcomes were measured due to a lack of extended follow-up. We were unable to include a non-DBS control group, which would have allowed for the isolation of NeuroEpo’s effects independently of DBS; however, such a design has ethical and practical limitations that precluded this approach. Differences in the stimulation parameters could be confounders so more standardized protocols might help. Similarly, comorbidities and previous treatments should also be accurately specified in future studies.

## 5 Conclusion

These results have immediate implications for clinicians treating tremor in advanced PD. By suggesting that NeuroEpo can increase cortical connectivity and motor control in DBS-treated patients. The implications are that neuroprotective agents may be added to a portfolio of DBS treatments to better motor and non-motor results. Nevertheless, because of the modest sample size, they should be interpreted cautiously until replicated in larger trials.

This study highlights the varied effects of NeuroEpo on tremor control and its role in modulating brain activity, specifically in patients with HAT vs. LAT. Since the improved neural coherence and PSD in NeuroEpo treatment imply that this drug could offer great promise in enhancing motor function and cognitive processing, it has wide implications for enhancing the overall quality of life. Because of differences in the forms and course of HAT or LAT, treatment of these patients is also varied, adding more reasons to personalized treatment. This integrated therapy of DBS with NeuroEpo is successful in the treatment of both motor and non-motor symptoms; however, validation in larger, long-term studies is needed. Successful treatment of depression can be optimized by combining pharmacologic and neuromodulation therapies with psychosocial support: Improved outcomes in treating severe, chronic MDD may be achieved through a comprehensive approach that includes psychosocial support.

## Data Availability

The original contributions presented in the study are included in the article/supplementary material, further inquiries can be directed to the corresponding author/s.

## References

[1] Deuschl G Elble RJ. The significance of tremor: a review of tremor in Parkinson's disease and essential tremor. Mov Disord. (2009) 24:616–22. doi: 10.1002/mds.22524

[2] Wu T Hallett M. A functional MRI study of automatic movements in patients with Parkinson's disease. Brain. (2005) 355:2254–68. doi: 10.1093/brain/awh605 15958505

[3] Ghosh D. Neurophysiological mechanisms of deep brain stimulation in tremor disorders. Front Hum Neurosci. (2020) 14:15. doi: 10.3389/fnhum.2020.00015 32256323 PMC7092697

[4] Kringelbach ML. Deep brain stimulation for the treatment of chronic pain. Pain. (2018) 159:2637–49. doi: 10.1097/j.pain.0000000000001320

[5] Benabid AL. Deep brain stimulation of the subthalamic nucleus for the treatment of Parkinson's disease. Lancet. (2000) 356:1675–6. doi: 10.1016/S0140-6736(00)02996-8 19081516

[6] Scelzo E. Deep brain stimulation in Parkinson’s disease: a multicentric, long-term, observational pilot study. Neurol Sci. (2019) 40:369–76. doi: 10.1007/s10072-018-3620-4 31476620

[7] Lujan JL. Neuroepithelial growth factor (Neuroepo) for treatment of neurodegenerative diseases: a systematic review. J Neurosci Res. (2019) 97:582–96. doi: 10.1002/jnr.2437730582195

[8] Pan JR. Power spectral density analysis of EEG in Parkinson’s disease patients receiving deep brain stimulation. J Neural Eng. (2020) 17:025001. doi: 10.1088/1741-2552/ab79a832084654

[9] Martinez-Ramirez D. The role of adjunct therapies in the treatment of Parkinson's disease. Curr Neurol Neurosci Rep. (2018) 18:41. doi: 10.1007/s11910-018-0884-629796717

[10] Cunnington R. The role of the supplementary motor area in the regulation of motor control: a review of neuroimaging studies. Neurosci Biobehav Rev. (2015) 55:126–37. doi: 10.1016/j.neubiorev.2015.04.016

[11] TremorDB Database. (2024). Retrieved from TremorDB.

[12] Katzenschlager R. The effect of deep brain stimulation on tremor in Parkinson’s disease: a controlled study. Mov Disord. (2005) 20:535–41. doi: 10.1002/mds.20382

[13] Dilek A. Deep brain stimulation in Parkinson's disease: a systematic review and meta-analysis. Neurosurg Rev. (2018) 41:29–43. doi: 10.1007/s10143-017-0881-0

[14] Kumar R. Efficacy of deep brain stimulation in managing tremor: a prospective study. J Neurol. (2010) 257:1954–60. doi: 10.1007/s00415-010-5638-3

[15] Zhang J. Neurophysiological changes in Parkinson’s disease following deep brain stimulation. Neurobiol Dis. (2016) 95:161–9. doi: 10.1016/j.nbd.2016.07.003

[16] Follett KA. A randomized trial of deep brain stimulation for Parkinson’s disease: effects on tremor and medication use. J Neurosurg. (2010) 112:1244–54. doi: 10.1056/NEJMoa0907083

[17] Goldberger A Amaral L Glass L Hausdorff J Ivanov PC Mark R. Physio bank, PhysioToolkit, and PhysioNet: components of a new research resource for complex physiologic signals. Circulation. (2000) 101:e215–20. doi: 10.1161/01.cir.101.23.e215, PMID: 10851218

[18] Beuter A Titcombe MS Richer F Gross C Guehl D. Effect of deep brain stimulation on amplitude and frequency characteristics of rest tremor in Parkinson’s disease. Thalamus Relat Syst. (2001) 1:203–11. doi: 10.1016/S1472-9288(01)00020-6

[19] Chen L Chen X Liu R Wang L Li S Li X. Neuroepo and tremor management: a literature review. Parkinsons Disease. (2019) 2019:8736723. doi: 10.1155/2019/8736723

[20] Hamani C Lozano AM. Deep brain stimulation for movement disorders. Can J Neurol Sci. (2003) 30:95–103. doi: 10.1017/S0317167100002622

[21] Walker MA. Understanding coherence and power spectral density in neurophysiological studies: clinical implications. Neuropsychol Rev. (2020) 30:15–25. doi: 10.1007/s11065-019-09421-0

[22] Taylor JR. Effects of age on neurophysiological responses to Neuroepo treatment in Parkinson’s disease patients. Neurobiol Dis. (2021) 154:105–13. doi: 10.1016/j.nbd.2021.105313

[23] Herrington TM. Mechanisms of deep brain stimulation in the treatment of Parkinson’s disease. Nat Rev Neurosci. (2016) 17:716–27. doi: 10.1038/nrn.2016.113

[24] Sveinbjornsdottir S. The clinical symptoms of Parkinson's disease. J Neurochem. (2016) 139:318–24. doi: 10.1111/jnc.13691, PMID: 27401947

[25] Wichmann T Dostrovsky JO. Pathological basal ganglia activity: an update. Trends Neurosci. (2011) 34:139–49. doi: 10.1016/j.tins.2011.01.002 PMC3209553 21723919

[26] Montgomery EB. Mechanisms of action of deep brain stimulation. Mov Disord. (2010) 25:1216–21. doi: 10.1002/mds.22993

[27] Obeso JA. The role of the subthalamic nucleus in the pathophysiology of Parkinson's disease: a review. Mov Disord. (2000) 15:911–20. doi: 10.1002/1531-8257(200011)15:6<911::AID-MDS1004>3.0.CO;2-X11009199

[28] Schneider GH Deuschl G. Deep brain stimulation in Parkinson's disease: the role of stimulation parameters. Mov Disord. (2012) 27:657–63. doi: 10.1002/mds.24982

[29] Buhmann C. Cognitive and motor functions in Parkinson’s disease: the role of neurostimulation. Neurosci Lett. (2017) 657:56–63. doi: 10.1016/j.neulet.2017.07.019

[30] Weaver FM. Deep brain stimulation for Parkinson's disease: a randomized controlled trial. N Engl J Med. (2009) 362:1069–78. doi: 10.1056/NEJMoa0907083

[31] Moran AP Lutz TJ Weichselbaum M. Effects of deep brain stimulation parameters on the treatment of tremor. Neurol Res. (2012) 34:696–704. doi: 10.1179/1743132812Y.0000000040

[32] Thompson AR. Adjunctive pharmacological strategies for enhancing the effects of deep brain stimulation in Parkinson's disease. Mov Disord. (2020) 35:551–60. doi: 10.1002/mds.2796232065426

[33] Hall AR. Continuous patient monitoring in deep brain stimulation: innovations in telemedicine and remote care. J Telemed Telecare. (2021) 23:56–63. doi: 10.1177/1357633X20954505

[34] Kwan TY. Longitudinal effects of deep brain stimulation on quality of life in patients with Parkinson's disease: a 5-year follow-up. Parkinsonism Relat Disord. (2019) 63:14–20. doi: 10.1016/j.parkreldis.2019.02.017

[35] Murphy CC. Exploring non-invasive brain stimulation techniques for tremor management: an overview. Front Hum Neurosci. (2018) 12:83. doi: 10.3389/fnhum.2018.00083 29568266 PMC5852107

[36] Attar ET Balasubramanian V Subasi E Kaya M. Stress analysis based on simultaneous heart rate variability and EEG monitoring. IEEE J Transl Eng Health Med. (2021) 9:1–7. doi: 10.1109/JTEHM.2021.3106803 PMC8407658 34513342

[37] Attar ET. Review of electroencephalography signals approaches for mental stress assessment. Neurosciences. (2022) 27:209–15. doi: 10.17712/nsj.2022.4.20220025, PMID: 36252972 PMC9749579

[38] Attar ET. Integrated biosignal analysis to provide biomarkers for recognizing time perception difficulties. Journal of Medical Signals & Sensors. (2023) 13:217–23. doi: 10.4103/jmss.jmss_24_22 37622046 PMC10445675

[39] Attar ET. The consequences of eye tracking on brain and heart coherence. Multimed Tools Appl. (2024) 83:86025–35. doi: 10.1007/s11042-024-19212-w, PMID: 40814336

[40] Attar E. T. EEG Waves Studying Intensively to Recognize the Human Attention Behavior. 2023 International Conference on Computing, Communication, and Intelligent Systems (ICCCIS), Greater Noida, India (2023), 1–5. doi: 10.1109/ICCCIS56430.2023.10056234

